# Lightweight Gypsum Materials with Potential Use for Thermal Insulations

**DOI:** 10.3390/ma13235454

**Published:** 2020-11-30

**Authors:** Cristina Dima, Alina Badanoiu, Silviu Cirstea, Adrian Ionut Nicoara, Stefania Stoleriu

**Affiliations:** Faculty of Applied Chemistry and Materials Science, University Politehnica of Bucharest, Str. Ghe. Polizu 1-7, 011061 Bucharest, Romania; dima_cryss@yahoo.com (C.D.); cirstea.silviu94@gmail.com (S.C.); adrian.nicoara@upb.ro (A.I.N.); stefania.stoleriu@upb.ro (S.S.)

**Keywords:** gypsum binder, additives, light-materials, porosity, thermal insulation

## Abstract

This article presents the influence of three additions i.e., hydroxyethyl methyl cellulose (HEMC), sodium bicarbonate and flue gas desulfurization (FGD) gypsum on the porosity of gypsum-based materials. The specific microstructure for a material with good thermal insulation properties i.e., numerous closed pores distributed in the binding matrix, was achieved using HEMC (0.3 wt.%) and sodium bicarbonate (0.5–2 wt.%). The addition of HEMC to the gypsum binder determines, as expected, an increase of the porosity due to its ability to stabilize entrained air. In the case of a sodium bicarbonate addition, the pores are formed in the binding matrix due to the entrapment of the gas (CO_2_) generated by its reaction. Sodium bicarbonate addition delays the setting of gypsum binder therefore in this study FGD gypsum (waste produced in the desulfurization process of combustion gases generated in power plants) was also added to the mixture to mitigate this negative effect. The decrease of geometrical density (up to 13%, in correlation with the additive nature and dosage) correlated with the increase of the porosity, determines, as expected, the decrease of flexural and compressive strengths (33–75%), but improves the thermal properties i.e., decreases the thermal conductivity (9–18%).

## 1. Introduction

Porous materials have numerous applications which depend on the composition of material, as well as number of pores, distribution, shape, size, connectivity, etc. [1]. Among the numerous applications of porous materials are thermal and acoustic insulations, filtration membranes, heavy metal absorption, catalysis, electro-magnetic interference shielding, energy and health (scaffolds, substrates for controlled drug release, wound healing, etc.) [1,2,3].

Porous materials used for thermal insulations are numerous and possess a wide range of properties. Nowadays, the most common thermal insulation materials are those based on organic compounds such as polyurethane and polystyrene; these organic materials have low densities and low values of thermal conductivity, but their main disadvantage is reduced fire resistance. Therefore, part of the research currently performed in this area focusses on the development of inorganic, fire-resistant thermal insulation materials [4,5,6]. Another topic related to the development of highly efficient and resistant thermal insulation materials is the incorporation of nanomaterials such as graphene oxide, nanocellulose or aerogels [2,7,8,9]. The high manufacturing cost of these nanomaterials is the main drawback which limits their current application on a large scale in the construction industry [9].

Another material which can be used for the development of cost-effective light weight porous materials with good thermal and acoustical insulation properties is gypsum [4,10].

Gypsum binders are obtained by the thermal treatment of gypsum rock (CaSO_4_·2H_2_O–CsD) at temperatures above 105 °C, when it can be transformed in hemi-hydrate (CaSO_4_·0.5H_2_O–CsH) or in anhydrite (CaSO_4_)—at higher temperatures [11]. Due to the relatively low thermal treatment temperatures and the possibility to recycle it in close loops, the gypsum binder is considered environmentally friendly as compared with other types of inorganic cements [12]. Nowadays, one of the main utilizations of gypsum binder is the production of gypsum plasterboards (dry-walls) [11,12]; another utilization of gypsum binder is to produce thermal and sound insulation materials [4,11,13,14,15,16,17,18]. According to Dolezelova et al. [10], gypsum-based light materials can be used in construction to replace autoclaved aerated concrete (AAC) and the energy consumption for the manufacture of gypsum materials is lower as compared with the one used for the manufacture of AAC.

The thermal insulation materials based on gypsum can be obtained either by its mixing with lightweight aggregates, including various waste such as expanded polystyrene [18], rubber, polyurethane foam and chopped electric cables waste [15,16], or direct foaming using various types of gas generators (aluminum sulphate with citric acid, calcium carbonate or sodium bicarbonate [4,13,14]). The thermal conductivity of this type of materials can vary from 0.085 up to 0.416 W/(m.K) depending on the nature and dosage of the additions and the assessment method [4,10,14,15]. 

In this paper gypsum-based materials were prepared using two foaming additions: hydroxyethyl methyl cellulose (HEMC) and sodium bicarbonate. In contact with water, sodium bicarbonate generates CO_2_, which can be trapped in the binding matrix, thus generating supplementary porosity. The presence of sodium bicarbonate in gypsum paste delays the binders setting, and so therefore in the materials presented in this paper, flue gas desulfurization gypsum (FGD gypsum) was used as a setting accelerator.

FGD gypsum is a waste produced in the desulfurization process of combustion gases generated in power plants. The desulphurization process of exhaust gasses from combustion plants is imposed by the EU legislative framework regarding emissions [19]. This process consists of SO_2_ (from flue gas) reaction with an alkaline substance (such as dolomite, limestone, lime, or hydrated lime) to produce sulphite or sulphate [20]. The presence of CaSO_4_·2H_2_O (CsD) as a main component in FGD gypsum justifies its use as a setting accelerator in gypsum binders. The CsD particles from FGD gypsum can act as nucleation sites for newly formed CsD crystals (by CaSO_4_·0.5H_2_O hydration), therefore accelerating the setting process [14,16].

Hydroxyethyl methyl cellulose addition was used to produce supplementary porosity, due to its ability to stabilize the air entrained during the mixing of the components (high affinity for the water-air interface) [21,22].

This paper presents the influence of these three additions (sodium bicarbonate, FGD gypsum and hydroxyethyl methyl cellulose) on the main properties of gypsum binders i.e., setting time, geometrical density, open porosity, compressive strength, and thermal conductivity.

## 2. Materials and Methods

**Figure 1:**
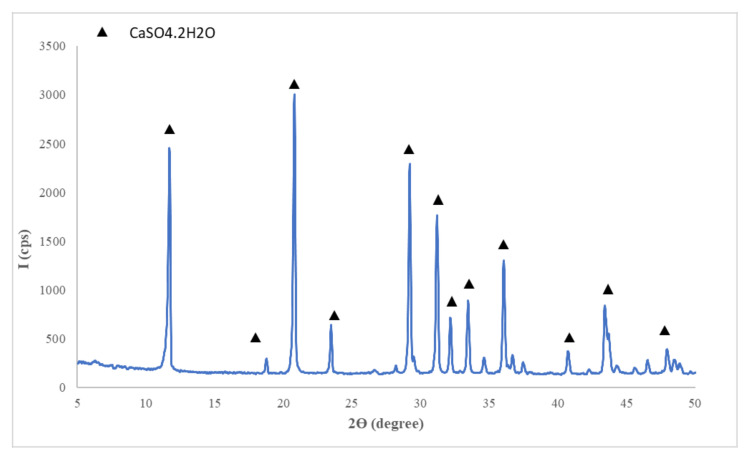
XRD patterns of flue gas desulfurization (FGD) gypsum.

The materials used in this research were:

- Gypsum binder (Saint-Gobain, Bucharest, Romania) with the following characteristics: calcium sulphate content over 50%, setting time 15-20 min and a fineness corresponding to 99% passing through 315 µm sieve;
- Flue gas desulfurization (FGD) gypsum (CET, Romania) resulted in the desulfurization process of gases emitted during coal combustion in a power plant; the as-received FGD gypsum was moist (approx. 40% water) therefore it was dried at 40 °C and grinded up to a fineness corresponding to 98% passing through 200 µm sieve, before mixing with the other components; the main compound assessed by X ray diffraction (XRD) in FGD gypsum is CaSO_4_·2H_2_O (CsD)—Figure 1.
- Sodium bicarbonate (NaHCO_3_) (Sigma-Aldrich, Darmstadt, Germany) chemical reagent;
- Hydroxyethyl methyl cellulose (HEMC) (Dow, Midland, MI, USA)—chemical product.

The composition of studied materials is presented in Table 1.

The compositions were labelled as follow: I—gypsum binder; G—FGD gypsum (1 wt.%, 3 wt.%, 5 wt.% or 10 wt.%); B—sodium bicarbonate (0.5 wt.%, 1 wt.% or 2 wt.%); C—HEMC (0.3 wt.%).

For all compositions, the water to binder ratio was 0.6.

The following analysis were performed on binder pastes:

- Setting times were assessed according to the methods presented in ASTM C 472—99 and GB/T17669.4-1999 [23,24];
- Compressive or/and flexural strengths were assessed on paste specimens (cubes—20 × 20 × 20 mm and cuboids—40 × 40 × 160 mm) hardened for 7 days in air; the cuboid specimens were dried at 40 ± 2 °C up to constant mass before the test; minimum 3 flexural strength values and minimum 6 compressive strength values were considered for the calculation of average values of flexural and compressive strengths; the mixing procedure for paste preparation, the curing conditions (air, RH = 50 ± 5%, 23 ± 2 °C) and the procedure for mechanical testing are described in EN 13279-2:2014 [25];
- Geometrical density was assessed on cubes (20 × 20 × 20 mm) and was calculated as mass to volume ratio based on minimum 5 values (assessed on specimens cured in similar conditions);
- Open porosity was measured on fractured specimens, using the liquid saturation method under vacuum [26]; the working liquid was xylene (ρ = 0.866 g/mL).
- Thermal conductivity was assessed according to European Standard EN 12667 [27] on boards (300 × 300 × 20 mm) hardened for 7 days in air (RH = 50 ± 5%, 23 ± 2 °C). Before testing the specimens were dried at 40 °C up to constant mass. The HESTO-Lambda-CONTROL A90 (HESTO Elektronik GmbH, Steinbach, Germany) equipment measures the heat flow through a specimen placed between two plates with different temperatures [27]. All tests were performed at 23 ± 2°C and the values at 10 °C were calculated with the equipment’s software.
- X ray diffraction analyses were performed on a Shimadzu XRD 6000 (Shimadzu, Kyoto, Japan) with monochromatic radiation CuKα (λ = 1.5406 Å);
- Scanning Electron Microscopy (SEM) analyses were performed on pastes hardened in air, using a high-resolution electronic scanning microscope equipped with a Schottky emission electron beam FEI Inspect F50 (Thermo Fisher—former FEI, Eindhoven, Nederland) with a resolution of 1.2 nm at 30 kV and 3 nm at 1 kV (BSE). All the SEM images were acquired on freshly fractured samples, fixed with carbon tape on an aluminium holder, covered with gold by metallization for 45 s, and then visualized in a vacuum using a 30 kV acceleration voltage and spot 3.5.

## 3. Results and Discussion

**Figure 2:**
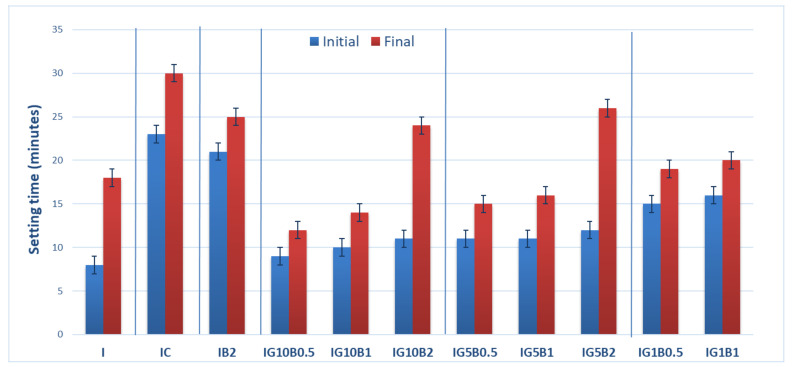
Setting times of gypsum binders with 0.3% HEMC (IC) and various amounts of sodium bicarbonate (0.5%, 1% and 2%) and FGD gypsum (1%, 5% and 10%).

The setting times of the compositions based on gypsum binder are presented in Figure 2. As can be seen, the addition of both HEMC and sodium bicarbonate strongly delay the setting times. The delay caused by the HEMC addition could be due to the formation of a polymer film on the surface of gypsum plaster grains which prevents their hydration.

The presence of sodium bicarbonate addition also inhibits the hydration of gypsum binder and delays the setting time; these data are in good correlation with those reported by Umponpanarat and Wansom [14].

To mitigate this effect FGD gypsum was added to the binding system. Previous results reported by our research group showed an important decrease of setting time when FGD gypsum is used as addition to gypsum binder [16]. The calcium sulphate dihydrate (CsD) crystals present in FGD gypsum can act as nucleation sites for the new CsD crystals, formed by the hydration of calcium sulphate hemihydrate (CsH) from gypsum plaster, therefore shortening the setting time (Figure 2).

As can be seen in Figure 2, the decrease of sodium bicarbonate (B) dosage from 2% up to 0.5% in the compositions with FGD gypsum (1%, 5% and 10%), decreases the setting times. The increase of FGD gypsum dosage from 5% up to 10% decreased the setting times.

**Figure 3:**
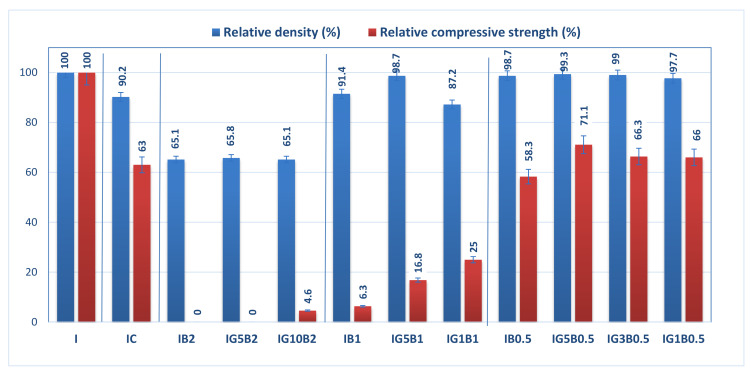
Influence of FGD gypsum (G), sodium bicarbonate (B) and HEMC (C) additions on the normalized geometrical density and compressive strength.

The relative geometrical density and relative compressive strength (calculated with reference to the values obtained for I specimen) for the gypsum binder with 0.3% HMEC and various amounts of sodium bicarbonate and FGD gypsum are presented in Figure 3.

The addition of HEMC to the gypsum binder determines an increase of the porosity (as will be further presented), and so therefore the values of geometrical density and compressive strengths are smaller for IC as compared with I.

The addition of sodium bicarbonate also decreases the geometrical density and compressive strengths in correlation with its dosage (Figure 3); for the highest dosage of sodium bicarbonate (2 wt.%) the compositions IB2 and IG5B2 have lower values of geometrical density (as compared with I), but no recordable compressive strength. The increase of FGD gypsum dosage up to 10 wt.% (IG10B2) determines a small increase of compressive strength values for a similar value of geometrical density.

As expected, the decrease of sodium bicarbonate dosage determines the increase of compressive strengths correlated with higher values of geometrical density. This evolution is in good correlation with the porosity of these materials (as will be presented further).

**Figure 4:**
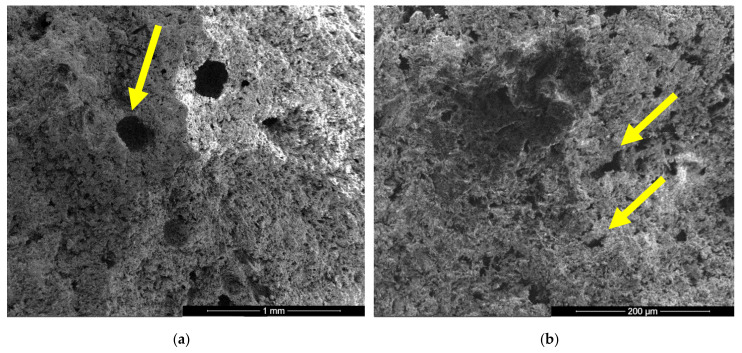
SEM images of gypsum binder (I) after 7 days of hardening in air, with various magnifications: (**a**) ×100; (**b**) ×500.

**Figure 5:**
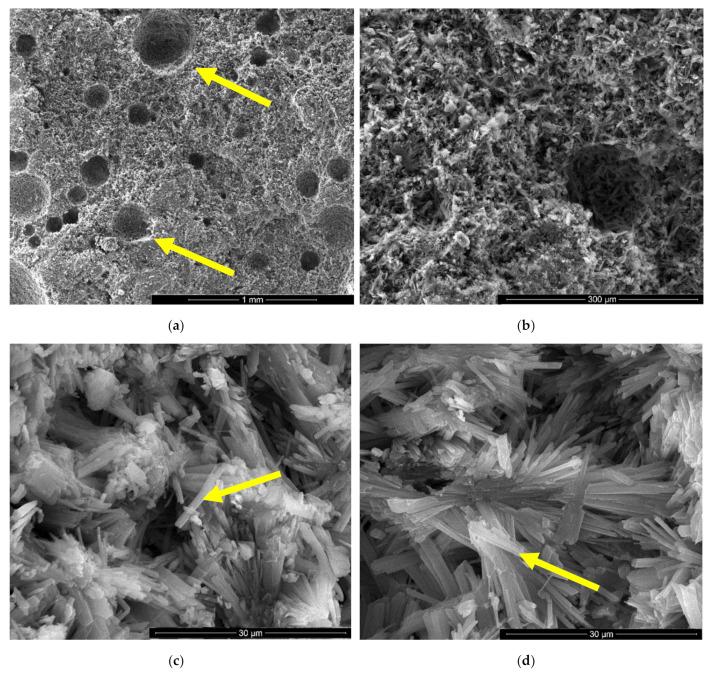
SEM images of gypsum binder with HEMC (IC) after 7 days of hardening in air with various magnifications: (**a**) ×100; (**b**) ×500; (**c**) ×5000; (**d**) ×5000.

**Figure 6:**
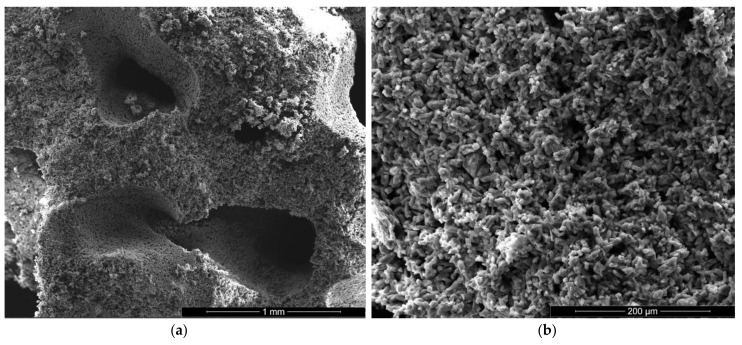

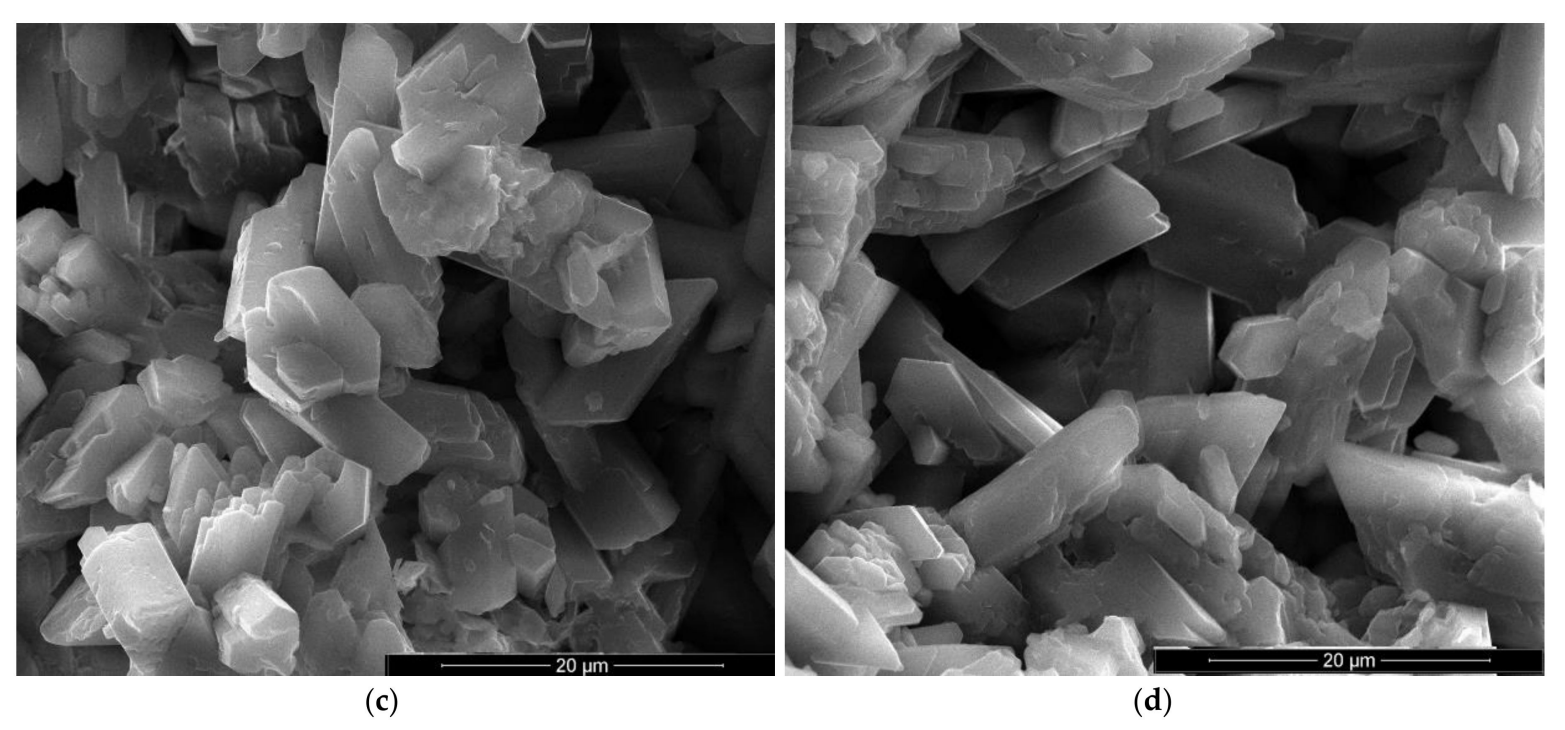
SEM images of gypsum plaster with 2% sodium bicarbonate addition (IB2) with various magnifications: (**a**) ×100; (**b**) ×500; (**c**) ×5000; (**d**) ×5000.

**Figure 7:**
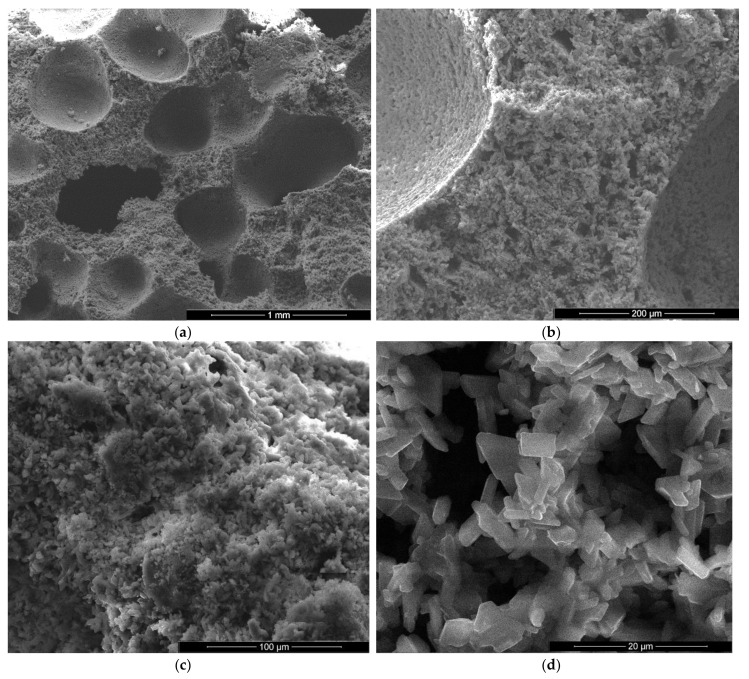
SEM images of gypsum plaster with 5% FGD and 2% sodium bicarbonate addition (IG5B2) with various magnifications: (**a**) ×100; (**b**) ×500; (**c**) ×1000; (**d**) ×5000.

**Figure 8:**
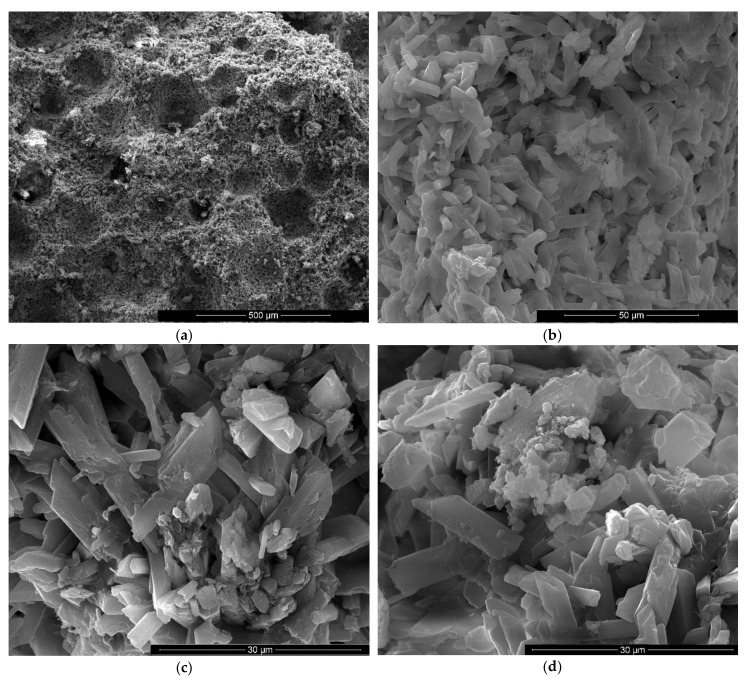
SEM images of gypsum binder with 1% FGD and 1% sodium bicarbonate addition (IG1B1) after 7 days of hardening in air with various magnifications: (**a**) ×200; (**b**) ×2000; (**c**) ×5000; (**d**) ×5000.

**Figure 9:**
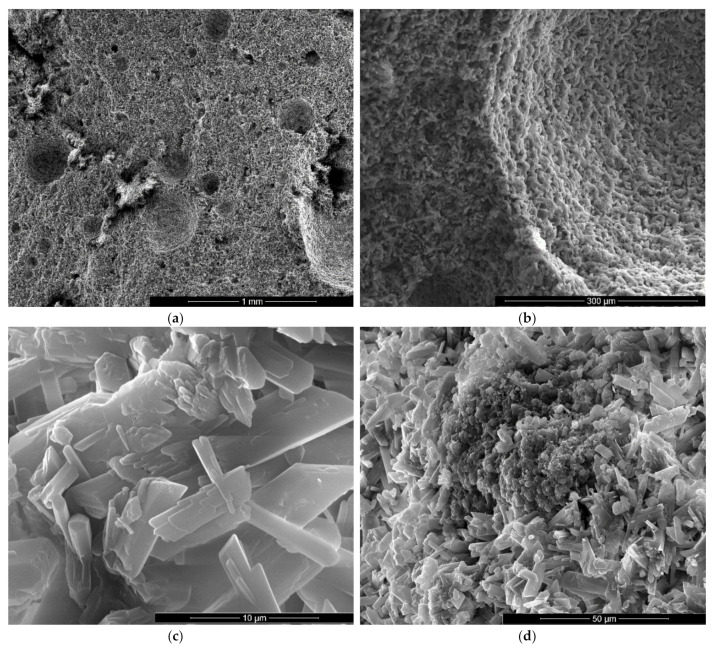
SEM images of gypsum binder with 1% FGD and 0.5% sodium bicarbonate addition (IG1B0.5) after 7 days of hardening in air, with various magnifications: (**a**) ×100; (**b**) ×500; (**c**) ×10000; (**d**) ×2000.

The microstructure of gypsum pastes hardened for 7 days in air was assessed by scanning electron microscopy (Figure 4, Figure 5, Figure 6, Figure 7, Figure 8 and Figure 9).

In the SEM images of I specimen one can notice the presence of large round pores with sizes comprised between 0.1–0.25 microns (see arrow in Figure 4a) formed by the air entraining during the mixing operation. In Figure 4b one can notice the presence of smaller pores with various shapes and sizes (see arrows) specifically for the binding matrices formed by the hydration of CsH; the long CsD crystals are interlocked and forms the binding matrix [13,15,28].

For the IC specimen (Figure 5) the presence of HEMC addition determines an important increase of pores numbers and sizes, up to 0.75–1 mm. A close-up on the binding matrix reveals the presence of interlocked needle-like CsD crystals specific for hardened gypsum binder (see arrow in Figure 5c,d). The increase of the number of round closed pores assessed on SEM images (see arrows in Figure 5a) when HEMC is added in the binding system, can be explained by its specific ability to stabilize the entrained air (high affinity for the water-air interface [21,22]).

For the specimens with sodium bicarbonate an important increase of volume was noticed shortly after the pouring of the paste in the mold. This phenomenon is due to the CO_2_ generation in the reaction of sodium bicarbonate with water and calcium sulphate hemihydrate (CsH) [13]. This process is more intense for a higher dosage of sodium bicarbonate and the resulted porosity is higher, as can be noticed for the SEM images presented in Figure 6, Figure 7, Figure 8 and Figure 9.

For the gypsum plaster with 2% sodium bicarbonate (IB2), one may assess in the SEM images the presence of big pores (over 1 mm) formed in the binding matrix by the released CO_2_ gas (Figure 6a); the irregular shapes of these pores suggest the coalescence of smaller pores.

It is interesting to note the shape of crystals in IB2 i.e., short thick crystals and plate like crystals (Figure 6c,d). The modification of CsD crystal shape and size can be due to the modification of reaction conditions in the presence of sodium bicarbonate addition [28] or/and to an oriented growth of crystals due to selective adsorption of retarding addition [24].

In the SEM images of plaster with 5% FGD gypsum and 2% sodium bicarbonate (IG5B2) one may notice the presence of numerous round pores with sizes comprised between 0.25–1 mm (Figure 7a) as well as smaller pores (10–20 microns) formed between the CsD crystals (Figure 7b,d). Short and plate-like CsH crystals also present in this composition (Figure 7c,d).

As expected, the reduction of sodium bicarbonate content reduces the average size of pores assessed on SEM images (Figure 8a and Figure 9a). The CsD crystals continues to be shorter with an average size of 10–20 microns. The interlocking of gypsum crystals increases (Figure 9b,c) and this contributes to the increase of mechanical strengths.

**Figure 10:**
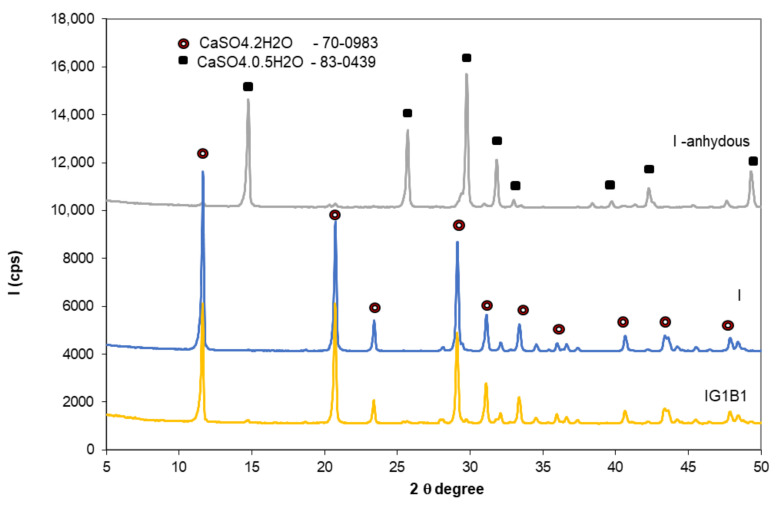
XRD patterns of gypsum binder I—anhydrous and I and IG1B1 hardened pastes.

Rubio Avalos et al. [13] reported the presence of Na_2_SO_4_ as secondary phase in the CaSO_4_·0.5H_2_O–NaHCO_3_–H_2_O system; according to these authors, the sodium sulphate (small round crystals) precipitates inside the gypsum crystals bulk. This phase was not detected in this study by XRD in the specimens with 0.5% and 1% sodium bicarbonate (Figure 10); this could be due to the low dosage of sodium bicarbonate in these compositions.

**Figure 11:**
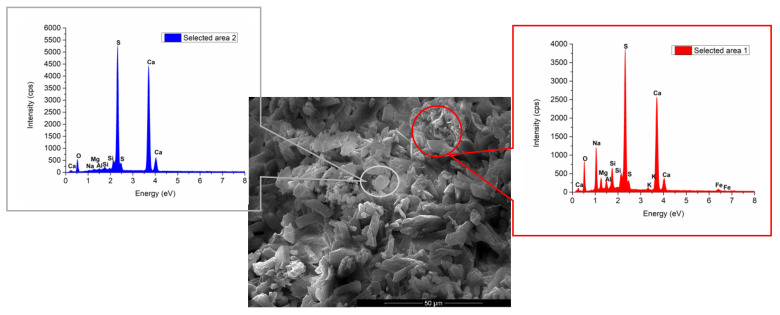
SEM and EDX analyses of IG1B1 paste.

Nevertheless, the EDX analysis presented in Figure 11 shows the presence of sodium in high quantity in some specific areas, which could be associated to a phase with sodium content.

The European norm EN 13279-1 sets the requirements for gypsum binders and plasters; these requirements refer to the flexural strength (higher than 1 N/mm^2^) and compressive strength (higher than 2 N/mm^2^) [29].

The compositions IC and IG1B1 fulfill the above-mentioned requirements (Table 2), and therefore thermal conductivity was assessed on these specimens. The thermal conductivity at 10 °C (set I), assessed in accordance with the norm EN ISO 10456 [30], is the thermal conductivity value usually declared by the European producers of this type of construction material based on the fact that 10 °C is considered the average yearly temperature at which the thermal insulation of buildings must operate.

As expected, the decrease of the geometrical density and increase of open porosity of IC and IG1B1, due to the presence of hydroxyethyl methyl cellulose (HEMC) and sodium bicarbonate, improves the thermal properties of these materials (i.e., reduces the values of thermal conductivity at 10 °C).

In conformity with European norm EN 13279-1 [29], gypsum plasters or gypsum binders are classified as reaction to fire Class A1 when they contain less than 1% by weight or volume of organic materials, without supplementary testing. The only material presented in this paper which contains organic material is the one with hydroxyethyl methyl cellulose but its amount (0.3%) is below the previously mentioned limit; therefore, the gypsum based materials obtained in this study can be classified as Class A1 (reaction to fire).

Based on the properties assessed for the studied gypsum-based materials a potential practical application could be for the manufacture of light gypsum blocks/boards for non-load-bearing walls with improved thermal insulation properties and good fire behavior.

## 4. Conclusions

In this study light gypsum-based materials were obtained using hydroxyethyl methyl cellulose (HEMC) and sodium bicarbonate. To reduce the setting time of gypsum binders with sodium bicarbonate addition, FGD gypsum was also added to the mixture.

The following conclusions can therefore be drawn:

- The porosity of the gypsum pastes with HEMC increases due to its specific ability to stabilize the entrained air during the mixing of solid and liquid components (high affinity for the water-air interface); the average size of the pores assessed by scanning electron microscopy (SEM) in these materials is comprised between 0.25–0.75 mm.
- The density of the materials with sodium bicarbonate decreases with the increase of NaHCO_3_ dosage. Numerous pores (assessed by SEM) are formed in the binding matrix due to the entrapment of the gas (CO_2_) generated by the NaHCO_3_ reaction. For the specimens with 0.5% and 1% sodium bicarbonate, pores with sizes comprised between 10 microns and 1 mm are assessed by SEM. For the compositions with 2% NaHCO_3_, big pores (sizes over 1 mm) with irregular shapes are present in the material; the irregular shapes of these pores suggest the coalescence of smaller pores.
- As expected, the decrease of density correlated with the increase of porosity determines the decrease of flexural and compressive strengths, with 37% for IC and 12–60% (flexural strength) and 33–75% (compressive strength) for the materials with sodium bicarbonate and FGD gypsum additions. Nevertheless, for an adequate dosage of the additives, flexural strengths higher than 2 N/mm^2^ and compressive strength higher than 5 N/mm^2^ were achieved.
- The values of thermal conductivity of the gypsum binders with the studied additives, decreases (9–18% with reference to plain gypsum). Moreover, because these materials contain less than 1% by weight of organic materials, in conformity with European norm EN 13279-1 they can be classified as Class A1—reaction to fire.

## Figures and Tables

**Table 1:** Composition of studied materials.

Specimens	Dosage (wt.%)			
	Gypsum Binder (I)	FGD Gypsum (G)	NaHCO_3_ * (B)	HEMC * (C)
I	100	-	-	-
IC	100	-	-	0.3
IB0.5	100	-	0.5	-
IB1	100	-	1	-
IB2	100	-	2	-
IG5B0.5	95	5	0.5	-
IG5B1	95	5	1	-
IG5B2	95	5	2	-
IG10B0.5	90	10	0.5	-
IG10B1	90	10	1	-
IG10B2	90	10	2	-
IG3B0.5	97	3	0.5	-
IG1B0.5	99	1	0.5	-
IG1B1	99	1	1	-

* calculated with reference to I+G mixture; B and HEMC were added to I+G mixture.

**Table 2:** Geometrical density, open porosity, flexural and compressive strengths and thermal conductivity of gypsum-based materials.

Specimens	Geometrical Density (kg/m^3^)	Open Porosity (%)	Flexural Strength (N/mm^2^)	Compressive Strength (N/mm^2^)	Thermal Conductivity at 10 °C (W·m^−1^·K^−1^)
I	1253 ± 25	29.76 ± 0.30	5.35 ± 0.26	21.06 ± 1.05	0.2112 ± 0.0002
IC	1130 ± 22	32.87 ± 0.33	3.39 ± 0.15	13.25 ± 0.65	0.1917 ± 0.0002
IG1B1	1093 ± 21	37.38 ± 0.38	2.07 ± 0.05	5.26 ± 0.22	0.1736 ± 0.0002
